# Effect of Cellulose-Based Bioplastics on Current LDPE Recycling

**DOI:** 10.3390/ma16134869

**Published:** 2023-07-07

**Authors:** Giovanni Gadaleta, Sabino De Gisi, Andrea Sorrentino, Luigi Sorrentino, Michele Notarnicola, Kerstin Kuchta, Caterina Picuno, Maria Oliviero

**Affiliations:** 1 Department of Civil, Environmental, Land, Building Engineering and Chemistry (DICATECh), Politecnico di Bari, Via E. Orabona n. 4, I-70125 Bari, Italy; giovanni.gadaleta@poliba.it (G.G.); michele.notarnicola@poliba.it (M.N.); 2 Institute for Polymers, Composites and Biomaterials (IPCB), National Research Council (CNR), P.le E. Fermi n. 1, I-80055 Portici, Italy; luigi.sorrentino@cnr.it (L.S.); maria.oliviero@cnr.it (M.O.); 3 Circular Resource Engineering and Management, Hamburg University of Technology, Blohmstraße n. 15, D-21079 Hamburg, Germany; kuchta@tuhh.de; 4 DMTR Consulting SRL, Via G. Guevara 5, I-70124 Bari, Italy; caterina.picuno@dmtrconsulting.com

**Keywords:** bioplastic, cellulose acetate, mechanical recycling, LDPE, plastic waste

## Abstract

The increased use of bioplastics in the market has led to their presence in municipal solid waste streams alongside traditional fossil-based polymers, particularly low-density polyethylene (LDPE), which bioplastics often end up mixed with. This study aimed to assess the impact of cellulose acetate plasticized with triacetin (CAT) on the mechanical recycling of LDPE. LDPE–CAT blends with varying CAT content (0%, 1%, 5%, 7.5%, and 10% by weight) were prepared by melt extrusion and analyzed using scanning electron microscopy, Fourier-transform infrared spectroscopy, thermal analysis (thermogravimetric and differential scanning calorimetry), dynamic rheological measurements, and tensile tests. The results indicate that the presence of CAT does not significantly affect the chemical, thermal, and rheological properties of LDPE, and the addition of CAT at different levels does not promote LDPE degradation under typical processing conditions. However, the addition of CAT negatively impacts the processability and mechanical behavior of LDPE, resulting in the reduced quality of the recycled material. Thus, the presence of cellulose-based bioplastics in LDPE recycling streams should be avoided, and a specific sorting stream for bioplastics should be established.

## 1. Introduction

Several bioplastics have recently attracted considerable attention as possible alternatives to petroleum-based plastics. Even though the production of bioplastics worldwide is currently around 1% of total plastic production, this share is expected to increase significantly in the coming years [1]. As the use of bioplastics increases, they will inevitably end up in the municipal waste stream at the end of their life cycle [2]. All bioplastic items that comply with “industrial compostability” standards (as described by UNI EN 13432:2002, [3]) must be collected in the organic waste stream and treated by industrial anaerobic digestion and/or composting [4]. However, it should be noted that not all bioplastics are biodegradable and compostable. The term “bioplastics” is commonly used to describe a variety of materials that consist, at least partially, of bio-based (renewable) feedstock and/or are biodegradable. As a result, bioplastics can be divided into three categories: those that are both bio-based and biodegradable, those that are solely bio-based, and those that are only biodegradable. Poly (lactic acid) (PLA), poly (hydroxy alkanoates) (PHAs), and bio-based poly (butylene succinate) (bio-PBS), as well as plastics based on starch, cellulose, lignin, and chitosan, are some examples of bioplastics that are both bio-based and biodegradable. Bio-based poly (amides) (bio-PP), poly (ethylene) (bio-PE), and poly (ethylene terephthalate) (bio-PET) are examples of bioplastics that are bio-based but not biodegradable. Finally, poly (caprolactone) (PCL), poly (vinyl alcohol) (PVA), and poly (butylene adipate terephthalate) (PBAT) are examples of biodegradable bioplastics derived from fossil resources [5]. In turn, biodegradable bioplastics may or may not be compostable and in the latter case they should not be collected with organic waste streams. In addition, there is a significant lack of information about the degradation of some types of bioplastic waste by biological processes [6,7], which frequently results in users collecting bioplastics in the plastic waste stream [8]. This disposal route can have economic and environmental impacts that are not easily quantifiable [9,10]. While it may be technically feasible and convenient to collect all plastic packaging waste materials in a single stream, material recovery facilities (MRFs) may not be capable of effectively sorting and separating them into distinct homogeneous streams for conversion into secondary raw materials [11,12]. Unfortunately, MRFs typically do not provide a separate flow for bioplastic waste, which are, along with other difficult-to-recycle polymers, instead directed to the PLASMIX stream (mix of non-recyclable packaging and plastic waste) and ultimately incinerated for energy recovery [13]. Nonetheless, the increased presence of bioplastics in the PLASMIX stream should not have a significant impact on the performance of the process [14]. Regrettably, bioplastics can often become mixed with the more commonly recycled petroleum-based polymers, such as low-density polyethylene (LDPE), and then reprocessed to obtain a secondary raw material [15]. However, if the concentration of bioplastics exceeds a certain threshold, they can decrease the quality of the recycled plastic stream [8].

Nowadays, PLA, PHA, and PBAT are the most known and circulated bioplastics. Cellulose-based bioplastics account for 3.2% of global bioplastics production. However, their use is continuously growing due to significant improvements in the production process and the relative lowering of costs. Among the cellulose derivatives, cellulose esters are widely used for their good processability and excellent mechanical and thermal properties.

Cellulose acetate (CA) is the most important cellulose ester and is formally obtained from cellulose by the partial substitution of hydroxyl groups (OH) with acetyl groups (COCH_3_) through a process called “acetylation”.

The amount of acetyl groups introduced per number of monomers is indicated as the degree of substitution (SD) [16]. To further improve the processability of CA and increase its biodegradability, glycerol triacetate (triacetin) is usually used as a plasticizer [17]. Triacetin, as an ester of acetic acid and glycerol, is a good plasticizer of CA as it has the same functional groups (acetyl groups) [18]. The addition of triacetin enables the melt processing of cellulose acetate and improves its mechanical properties, to the point of meeting the application requirements of packaging [19]. Furthermore, by varying the SD, they can be melt processed in conditions close to that used for common polyolefins such as polypropylene (PP) and polyethylene (PE) [20]. CA is typically immiscible with most petroleum-based materials due to structural differences. Despite the poor compatibility between LDPE and CA, plasticizers normally used with CA can facilitate their blending, as previously shown in literature for cellulose acetate/low-density polyethylene fiber-reinforced composites [21]. However, the effect of varying levels of CA on the properties of LDPE is not evaluated in literature; also, some studies have reported a decrease in mechanical strength and ductility of LDPE with increasing content of others cellulose esters such as cellulose acetate butyrate cellulose and acetate phthalate [22,23]. These blends have been deemed acceptable for packaging applications in the food industry. Nevertheless, they did not consider the effect of plasticizers, did not account for the typical levels of cellulose ester found in plastic waste streams, and did not evaluate the possible degradation of LDPE during processing.

Therefore, the goal of this study was to assess the role of thermo-plasticized cellulose acetate in the mechanical recycling of LDPE. To achieve this, LDPE was blended with CA thermo-plasticized with triacetin (CAT) at various levels, reflecting the current plastic waste management practices, and processed through melt extrusion to simulate thermo-mechanical recycling processes. The effects of different CAT contents on the morphology, chemical structure, thermal, mechanical, and rheological properties of LDPE in the blends were then examined and compared with those of recycled neat LDPE.

## 2. Materials and Methods

### 2.1. Materials

GIBAPLAST (Varese, Italy) provided pellets of thermoplastic cellulose acetate (CAT) containing approximately 30% plasticizer content. CAT was composed of cellulose acetate (39.8 wt% acetyl content, degree of substitution (DS) 2.5, specific gravity ~1310 kg/m^3^, and average molecular weight M_n_~50,000) and triacetin (99.5% purity, molecular weight M_w_~218.2 g/mol). INEOS Olefins & Polymers Europe (Koeln, Germany) provided low-density polyethylene (LDPE) with a commercial name of 23H430, with a melt flow rate of 2.0 g/10 min, density of 923 kg/m^3^, and softening temperature of 95 °C.

### 2.2. Preparation of Recycled LPDE–CAT Blends and Films

**Figure 1:**
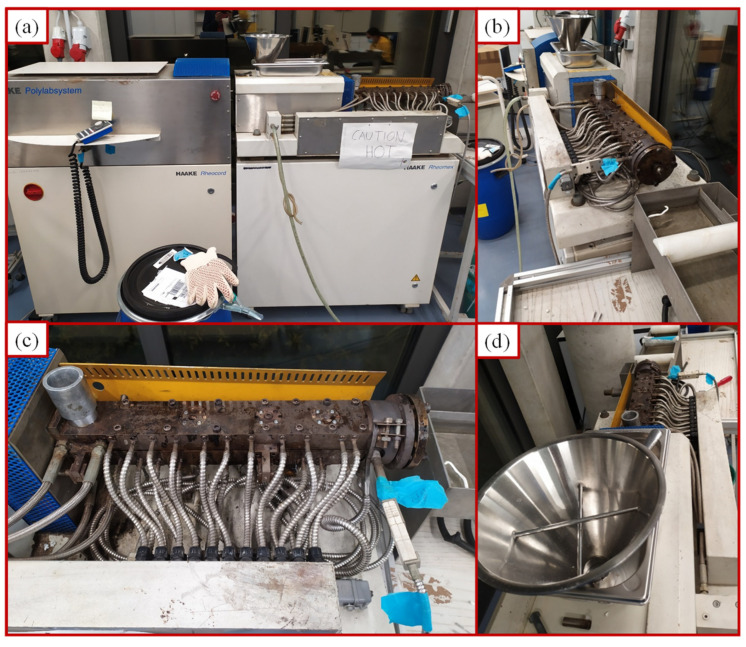
HAAKE Rheomex extruder (**a**), die zone (**b**), extrusion channel (**c**), and feed zone (**d**).

In order to assess the impact of CAT on the LDPE recycling process, LDPE was blended with different CAT contents (0, 1, 5, 7.5 and 10 wt%) and was subjected to an extrusion cycle to mimic themomechanical recycling. Before extrusion, LDPE and CAT were dried in an air-circulating oven at 60 °C for 1 day to remove the moisture absorbed during the storage. A HAAKE Rheomex CTW 100 OS (Thermo Fisher Scientific, Waltham, MS, USA) co-rotating twin screw extruder (L/D: 25/16), equipped with PolyLab Monitor software (v4.17) system (Figure 1), was used for extrusion all of the blends. The temperature profile was set at 155/160/165/170/175 °C (feed zone to die) to provide sufficient melting without the risk of further degradation. The screw speed was 100 rpm. To prevent overheating, a cooling air flow was used. The extruded strands with a diameter of about 5 mm were quickly cooled in a water bath to set the shape. Hereafter, the recycled blends are denoted as LDPE-xCAT, where x indicates the amount of CAT.

Films with a thickness of 0.3 mm were prepared using a P300P hot press (Collin, Germany). The extruded materials were heated and maintained at 180 °C and 50 bar for 10 min, then cooled under pressure to 30 °C. Specimens for various characterizations were cut from the central region of the films.

### 2.3. Characterization of Recycled LDPE–CAT Blends

Various analytical methods were employed to evaluate the morphological, chemical, thermal, mechanical, and rheological properties of the recycled LDPE-xCAT blends. These methods are detailed below.

#### 2.3.1. Scanning Electron Microscopy (SEM)

Scanning electron microscopy (SEM) images were taken with a SEM X Carl Zeiss Sigma 300 VP (Carl Zeiss Microscopy GmbH, Jena, Germany) operated at an acceleration voltage of 5 kV and at a working distance of 6–9 mm to observe the miscibility of the components in the blends subjected to a cryofracture process. Before observation, samples were coated with graphite by using Sputter Quorum Q150 (Quorum Technologies Ltd., Lewes, UK).

#### 2.3.2. Fourier-Transform Infrared Spectroscopy (FT-IR)

FT-IR analyses were performed using a Nicolet apparatus (Thermo Scientific, Italy) at ambient temperature to determine the effect of different amounts of CAT on the degradation of LDPE during the mechanical recycling process. The samples were analyzed in ATR mode from 4000 to 600 cm^−1^ with a wavenumber resolution of 4 cm^−1^ and an average of 64 scans.

#### 2.3.3. Thermogravimetric Analysis (TGA)

The thermal stability of neat LDPE and LDPE–CAT blends was determined by using a thermogravimetric analyzer TGA Q500 (TA Instruments, New Castle, DE, USA). The sample weight was 5 ± 1 mg. The samples were tested in a nitrogen atmosphere with a constant flow rate of 50 mL/min at temperatures ranging from 30 to 800 °C with a constant heating rate of 10 °C/min.

#### 2.3.4. Differential Scanning Calorimetry (DSC)

Thermal characterization of the neat LDPE and LDPE–CAT blends was carried out by using a differential scanning calorimeter (DSC Discovery, TA Instruments, New Castle, DE, USA). The samples were cut into small pieces with a weight of 5 ± 0.5 mg. The samples were first heated from −60 °C to 180 °C to eliminate their thermal history, then cooled to −60 °C and reheated to 180 °C at a constant rate of 10 °C/min under a nitrogen stream of 50 mL/min. All DSC experiments were performed in duplicate and the thermograms shown refer to the second heating and cooling scans. The degree of crystallinity, X_c_, of LDPE in each blend was determined from the relationship:

(1) $$X_{C}=\frac{\Delta H_{f}}{\Delta H_{f}^{0}}$$

where $\Delta H_{f}$ is the enthalpy of fusion determined from the thermogram and $\Delta H_{f}^{0}$ is the enthalpy of 100% crystalline LDPE material with a value of 277.1 J/g [24].

#### 2.3.5. Tensile Testing

The tensile properties of neat LDPE and LDPE–CAT blends were determined on universal testing machine SANS 4304 (Shanghai China) with a load cell of 10 kN. Samples with length and width of 100 mm and 16 mm, respectively, were cut from the produced films. The actual thickness was measured in at least three points for each sample, and the average value was used. Only samples with thickness variation lower than 5% were used for tensile testing. Tensile tests were performed according to ASTM D882-18 [25] and were carried out at room temperature with 50 mm as gauge length and 50 mm/min as crosshead speed. At least five experiments were performed for each sample and the average values were considered and discussed.

#### 2.3.6. Dynamic Rheological Measurement

The rheological properties of neat LDPE and LDPE–CAT blends were investigated using a RheoScope MARS II rheometer (HAAKE, Vreden, Germany) with parallel plates measuring 20 mm in diameter and a gap of approximately 0.2 mm. Circular specimens with a thickness of approximately 0.3 mm and a diameter of 20 mm were prepared for testing. The rheological measurements were conducted using a dynamic frequency sweep with a constant strain of 0.1% at 200 and 220 °C and included the determination of shear storage modulus (G′), shear loss modulus (G″), and complex viscosity (η∗) over a frequency range from 0.1 to 100 Hz.

## 3. Results

### 3.1. Morphological Analysis

**Figure 2:**
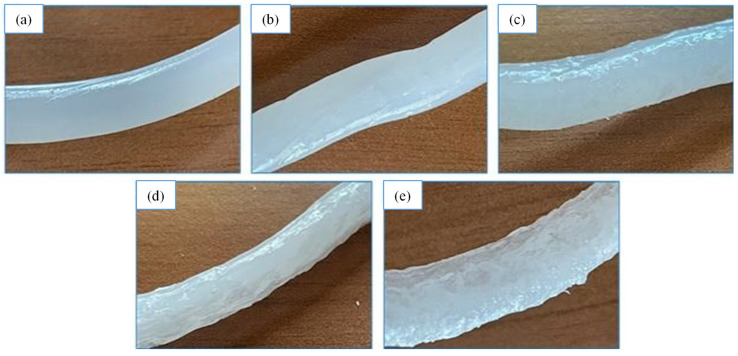
Visual inspection of extruded samples with varying CAT content: (**a**) 0%, (**b**) 1%, (**c**) 5%, (**d**) 7.5%, and (**e**) 10%.

Before investigating the changes in the processability of LDPE and the suitability for mechanical recycling, a visual inspection of the extruded samples was conducted. Figure 2 displays pictures of the various LDPE–CAT strands. The neat LDPE strands exhibited a smooth and clear surface morphology without significant irregularities (Figure 2a). However, the addition of CAT resulted in surface roughness and the sharkskin-like nature of the extruded strands, which became more apparent with increasing CAT content (Figure 2b–e). Although the immiscibility between the two polymers caused melt flow instabilities and the sharkskin-like effect caused the formation of various frays [26,27], the roughness can be mainly attributed to the presence of triacetin. During the extrusion process, triacetin, with a boiling temperature lower than the processing temperature of LDPE, evaporated but remained trapped within the polymer matrix, as highlighted by the presence of visible bubbles in the strands.

**Figure 3:**
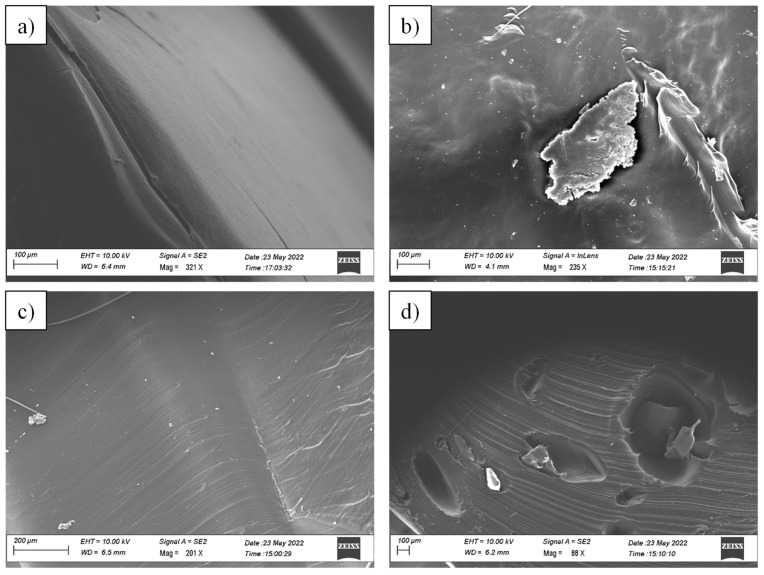
SEM images of: (**a**) surface of neat LDPE, (**b**) surface of LDPE-10CAT, (**c**) section of neat LDPE, and (**d**) section of LDPE-10CAT.

The microstructure of the extruded samples was examined using SEM. SEM analysis is essential for determining the morphological features of blends. It is commonly known that the weight ratio of the blending components primarily determines which of the two components created the matrix phase and the dispersed phase. In this case, the matrix phase represented the polymer with the largest proportion in the blend (i.e., LDPE) and the dispersed phase was the polymer CAT. Figure 3 shows the SEM images of the surface and fractured sections of both neat LDPE and the LDPE-10CAT blend. As anticipated, the surface of the samples appeared distinctly different, with neat LDPE (Figure 3a) being smooth and regular, while the surface of LDPE-10CAT displayed numerous inclusions and CAT particles (Figure 3b). In Figure 3d, the fracture surface of the LDPE-10CAT blend contained cavities and holes, whereas the neat LDPE fracture surface appeared smooth (Figure 3c). The presence of these cavities and holes indicated the evaporation of triacetin during the extrusion process and the incompatibility of LDPE and CAT, which created two distinct phases. Spherical domains of dispersed phases are more commonly formed in systems where phase separation occurs while the polymers are mixed in the molten state. The size of these domains provides information about the interactions between the blend components, in which bigger domains indicate poor interactions and smaller domains indicate better interactions [28]. The presence of these large holes formed during fracture meant that the weakly bound CAT dispersed phase was pulled out from the polyethylene matrix. This finding shows that there is no interaction between the CAT and LDPE phases. The fracture that occurred at the particle–matrix interface of the phases can be related to the weak interfacial adhesion between the two components. The lack of miscibility between these polymers also caused the uneven dispersion of CAT in the LDPE matrix and the non-homogenous fractured surface. This is consistent with the fact that LDPE is composed of a hydrophobic ethylene chain (non-polar polymer), while CAT has oxygen atoms with carbonyl functional groups (polar polymer), indicating its immiscible characteristics. Similar behavior has been reported by Sailaja [23] et al. for LDPE–cellulose acetate phthalate (CAP) blends. Additionally, in this case, the matrix underwent extensive shearing and cavitation with large holes left by the agglomerated CAP particles during the fracture. However, the authors reported that the presence of holes and the dimension of agglomerated particles can be reduced by the use of a suitable compatibilizer. Otherwise, it will possibly require a maleated LDPE to increase the compatibility of two phases, as established by Kosaka et al. [22] for a blend of LDPE and cellulose acetate butyrate (CAB).

### 3.2. FT-IR Spectroscopy

**Figure 4:**
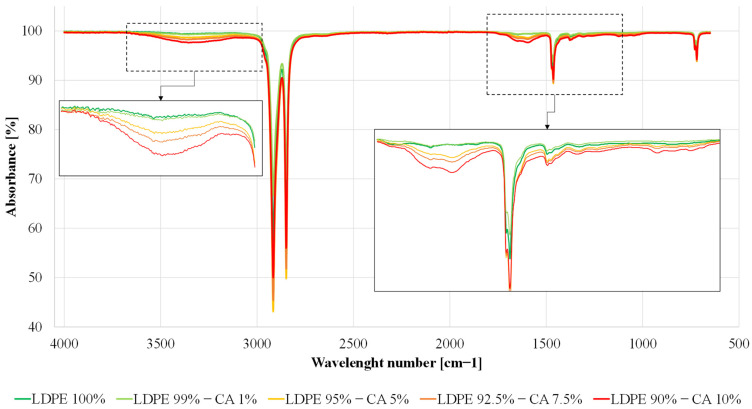
FT-IR spectra for neat LDPE and LDPE–CAT blends.

ATR-FTIR spectroscopy has been used as a useful tool both in determining specific groups or chemical bonds that exist in the developed materials and in evaluating the possible effect of CAT on LDPE degradation during mechanical recycling. For CAT and LDPE blends, if specific interactions took place between the two polymers, the most obvious and significant difference would be the appearance of new peaks or a shift in existing peaks. Figure 4 illustrates the ATR spectra of both neat LDPE and LDPE–CAT blends. The neat LDPE displayed characteristic peaks at wave numbers of 2915 cm^−1^, 2850 cm^−1^, 1460 cm^−1^, and 720 cm^−1^, corresponding to CH_2_ asymmetric stretching, CH_2_ symmetric stretching, bending deformation, and rocking deformation, respectively [29]. In addition to the previous peaks, the spectra of the LDPE–CAT blends show the typical peaks of cellulose acetate, including the -OH stretching of unacetylated cellulose, CH stretching of methyl groups (-CH_3_), carbonyl (C=O) stretching of acetate groups, H-O-H bending of absorbed water, CH_2_ bending, C-H bending vibration of CH_3_ in the acetyl group, C–O stretching of acetyl group, C–O–C stretching of cellulose backbone, and C-O-C stretching at β–(1→4) glycosidic linkages at 3483, 2947, 1738, 1642, 1437, 1373, 1211, 1033, and 901 cm^− 1^ m, respectively [30]. Additionally, the ATR spectra of blends show a new peak at 1556 cm^−1^ corresponding to the carboxylate group, which was initially absent in neat LDPE and CAT. However, this peak is not attributed to the formation of specific interactions between CAT and LDPE, but can be considered a consequence of thermal treatment (extrusion) of the LDPE in the blends, as reported by Chaudhary et al. [29]. LDPE in blends, having a greater amorphous phase, is more sensitive to thermal treatments than neat LDPE [31]. Generally, the formation of carbonyl (C=O) is indicative of polyethylene degradation. In the literature, the carbonyl index was calculated to investigate the degradation of recycled LDPE, based on the relative intensity of the carbonyl band at 1715 cm^−1^ to that of the methylene scissoring band at 1464 cm^−1^ [24]. In this case, the neat LDPE and blends had no peak formation at 1715 cm^−1^ after the recycling process. This suggests that there was no degradation of neat LDPE, and that the addition of CAT in various proportions did not encourage the degradation of LDPE after one cycle of extrusion. Similar results were obtained by Pedroso et al. for virgin LDPE. The authors instead reported a certain amount of polymer degradation after the extrusion of recycled LDPE. Probably, more cycles of extrusion must be considered to obtain partial degradation of LDPE. This behavior is desirable since the carbonyl groups present in recycled LDPE can promote better interaction with the polar groups of cellulose acetate [24].

### 3.3. Thermal Characterization

#### 3.3.1. Thermogravimetric Analysis

**Figure 5:**
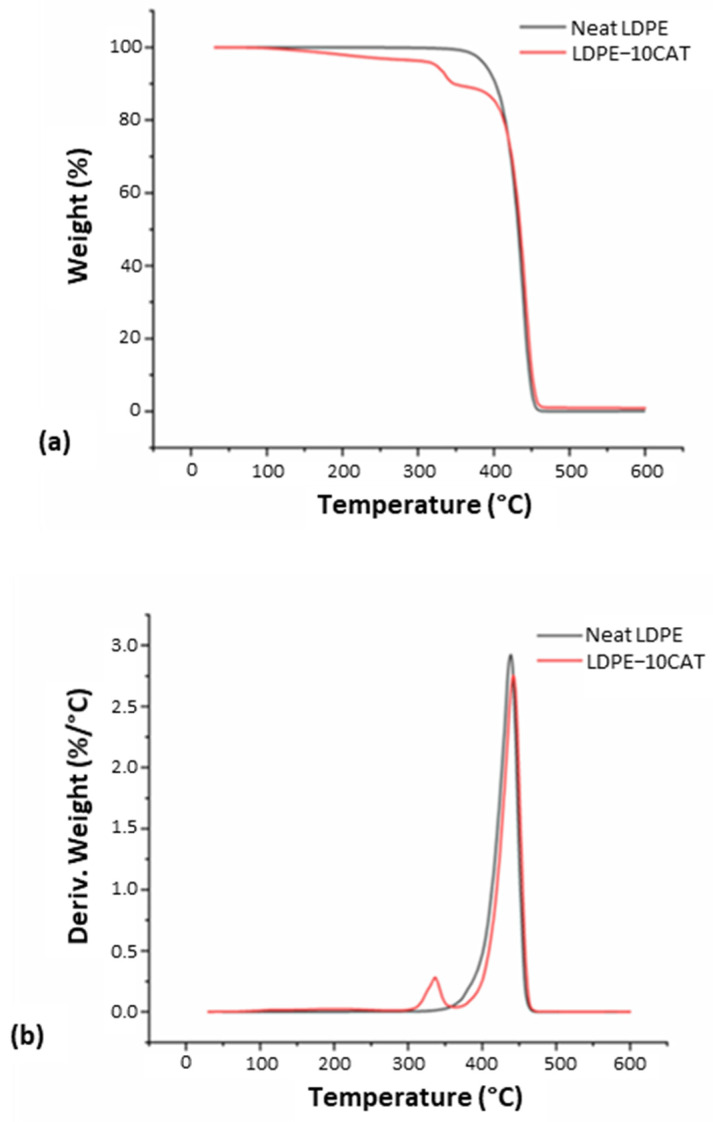
TGA (**a**) and DTG (**b**) curves for neat LDPE and LDPE-10CAT.

The weight loss curves for neat LDPE and LDPE-10CAT are shown in Figure 5a; the curves for other blends are not included for the sake of brevity. The TGA curve for neat LDPE displayed a single-stage degradation process at around 370–480 °C, which corresponds to the structural decomposition of the polymer [29]. It is noteworthy that the degradation of polyethylene is associated with the scission reactions of the C-C backbone caused by oxygen, which results in the formation of radicals that can decompose easily, generating oxidized species. After decomposition, neat LDPE exhibited almost 100% weight loss. In contrast, the TGA curve of the LDPE-10CAT blend indicated two distinct degradation stages, as expected for immiscible blends. The first stage at around 310–350 °C corresponded to the decomposition of CAT (i.e., breakage of glucosidic units), while the second stage at around 370–480 °C corresponded to the decomposition of LDPE [23]. Thus, the contents of CAT and LDPE phases in the blend could be estimated. The weight loss in the first degradation stage for LDPE-10CAT blend was approximately 7%, which roughly corresponds to the CAT content in the blend, also considering the evaporation of plasticizer during extrusion. In a previous study, the authors reported that the TGA curve of CAT shows two successive degradation steps: plasticizer evaporation at around 220 °C and thermal pyrolysis of the cellulose acetate backbone at around 369 °C [30]. However, after blending, the plasticizer evaporation step disappeared from the TGA curve, indicating the complete loss of triacetin from the LDPE-10CAT sample during the recycling process.

The temperature of maximum decomposition can be determined by identifying the peak degradation rate on the weight loss curve. Figure 5b displays the derivative of the weight loss curves, which allows for clear identification of the maximum decomposition temperature. The data in Table 1 reveal that LDPE has greater thermal stability compared to CAT. Blending with LDPE further lowered the thermal stability of CAT, indicating partial thermal degradation of the bioplastic during processing. In contrast, the thermal stability of LDPE slightly increased, suggesting a favorable interaction between the LDPE and biodegradable CAT material at their interface. We speculate that the LDPE–CAT molecular chains may have had some level of interaction that slowed down LDPE degradation. However, these interactions were likely not strong enough to have an impact on CAT stability.

#### 3.3.2. DSC Analysis

**Figure 6:**
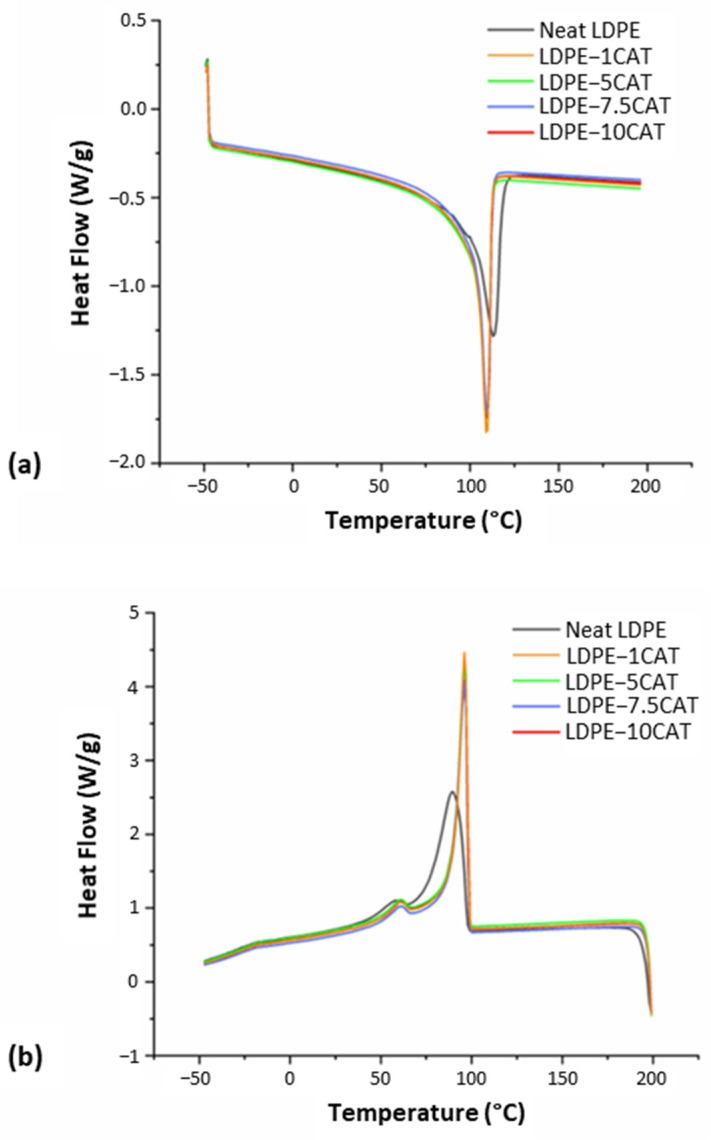
DSC thermograms of neat LDPE and LDPE–CAT blends: (**a**) heat and (**b**) cool.

The melting and crystallization behaviors of the neat LDPE and LDPE–CAT blends were investigated using DSC. The heating and cooling thermograms of neat LDPE and LDPE–CAT blends are shown in Figure 6a,b, respectively. From the thermograms, the corresponding average values of the melting temperature T_m_, crystallization temperature T_c1_, and relative crystallinity (determined from the enthalpy of fusion (Equation (1)) that is, ΔHc_1_ at T_c1_), were obtained and are summarized in Table 2. For neat LDPE, the values of T_m_ and T_c1_ were ~116 °C and ~90 °C, respectively, as indicated by the black lines on the thermogram plots in Figure 6 [32]. The LDPE–CAT blends showed deviations in the values of T_m_ and T_c1_ compared with neat LDPE. In particular, as shown in Figure 6, the T_m_ of LDPE in the LDPE–CAT blends shifted to a lower temperature, independently of the amount of CAT in the blends. This reduction may be due to the formation of imperfect crystallites caused by the presence of CAT [33]. Conversely, the T_c1_ of LDPE increased to the same value for all blends. This increase may be due to CAT chains in the blends retarding the crystallization of LDPE [34]. Aumnate et al. reported similar results for polypropylene/polyethylene blends, showing that the thermal properties were independent of the blend composition [35].

It is important to mention that the glass transition temperature (T_g_) of neat CAT is 127 °C, as previously reported [30]. However, no individual transition corresponding to T_g_ was observed in any of the LDPE–CAT thermograms shown in Figure 6a, suggesting that the loss of triacetin during the extrusion process caused CAT to return to its native form with a T_g_ of about 198 °C. This T_g_ value is too close to the upper limit of the temperature range considered in the DSC measurements.

All cooling curves in Figure 6b show evidence of a second broad crystallization transition, T_c2_, at approximately 58 °C for neat LDPE and 60 °C for all blends. The corresponding enthalpy of crystallization (ΔHc_2_ at T_c2_) for neat LDPE and the blends are summarized in Table 2. This second crystallization transition is commonly observed in LDPEs with low bulk crystallinity and is associated with the crystallization of thinner crystal lamellae at lower temperatures while thicker crystal lamellae are formed at higher temperatures [36]. As a result, different crystalline fractions are formed as the LDPE is re-crystallized from the melt.

The enthalpy change associated with the second crystallization transition is higher for neat LDPE and decreases with CAT addition. Therefore, the presence of CAT generally reduces the formation of thinner, less defined crystallites and results in a reduction in the overall bulk crystallinity as the wt% fraction of CAT increases (see Table 2). This highlights the fact that, at these wt% fractions of CAT, the molecules in the blend may hinder the crystallization of the LDPE. Comparable results have been reported by Heeley et al. [37] for PE–POSS blends. In particular, the authors reported that the effect of the dispersed phase on bulk crystallinity depends on wt% fractions of POSS. In particular, they indicated that POSS dispersed in the PE matrix at low levels may potentially act as a nucleating agent for the crystallization process, whereas high levels of POSS, comparable with levels of CAT in this work, suppress crystallization. The same trend was also observed by Kuang et al. [38] when they studied the effect of different contents of cellulose acetate on crystallization of polylactic acid.

### 3.4. Rheological Properties

**Figure 7:**
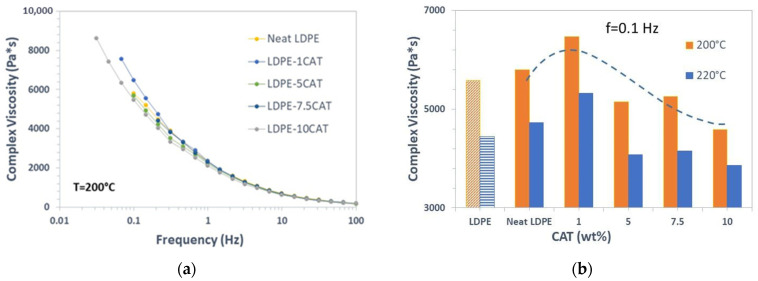
(**a**) Complex viscosity curves of neat LDPE and LDPE composites, (**b**) complex viscosity as function of CAT content at a frequency of 0.1 Hz (values from a commercial LDPE are represented with a lighter/striped pattern while the ones related to the tested LDPE are represented with the pattern as explained by the legend).

The measurement of flow curves within a wide range of shear rates and melt temperatures allow the collection of information necessary to evaluate the effect of mechanical recycling on rheological properties of the studied systems. The rheological properties of neat LDPE and LDPE–CAT blends were examined using oscillatory mode to measure storage modulus (G′), loss modulus (G″), and complex viscosity as a function of frequency. Figure 7a displays the complex viscosity curves at 200 °C of neat LDPE and LDPE blends, ranging from 0.1–100 Hz. The rheological data indicate a general trend of reduced complex viscosity with increasing frequency, which suggests shear-thinning behavior. This result can be attributed to the fact that at low and moderate shear rates, the long and flexible macromolecular chains of LDPE can entangle among themselves and also with CAT chains, hindering the flow of the melt and, consequently, increasing the viscosity. However, at high shear rates, LDPE macromolecular chains in the blends tend to disentangle and align, leading to a slippage between the chains and thus decreasing the viscosities [39]. This kind of trend is generally observed with blends made up of incompatible polymers as a result of the weakness at the planes between the interfaces [40]. In fact, different authors report this behavior for immiscible blends and nanocomposites; notably, we mention Djellali et al. [41] who analyzed the rheological properties of blends based on LDPE and PLA and Baghaei et al. [42] who instead have studied low-density polyethylene (LDPE)/ethylene–octene copolymer (POE)/organo-montmorillonite (OMMT) nanocomposites, prepared via melt compounding. In all these studies, as in our case, the presence of the dispersed phase has only a slight effect on viscosity, with a decrease observed as the content of this phase increases. The viscosity curves of the LDPE and blends superimpose at higher frequencies, with the difference between the 5 wt% and 10 wt% CAT becoming negligible for shear rates over 1 Hz. However, for shear rates below 0.1 Hz, the difference in viscosity remains significant. Figure 7b illustrates the complex viscosity of all systems at a frequency of 0.1 Hz measured at both 200 °C and 220 °C, along with the complex viscosity of unextruded LDPE. The data show that processing can cause degradation in LDPE, although it is quite resistant to degradation, and factors such as temperature, shear, and the presence of oxygen can lead to thermo-mechanical and thermo-oxidative degradation. In LDPE, both chain scission and crosslinking reactions can occur during extrusion, with crosslinking being more prevalent [43]. Therefore, the increase in viscosity after extrusion may be due to reduced polymeric chain mobility caused by crosslinking. The addition of CAT reduces composite viscosity, even at 220 °C, but at 1% concentration, an increase in viscosity is observed due to possible interactions between CAT and LDPE during extrusion. At higher CAT concentrations, the plasticization effect of CAT may be dominant over crosslinking due to thermal degradation of the composites.

### 3.5. Mechanical Properties

**Figure 8:**
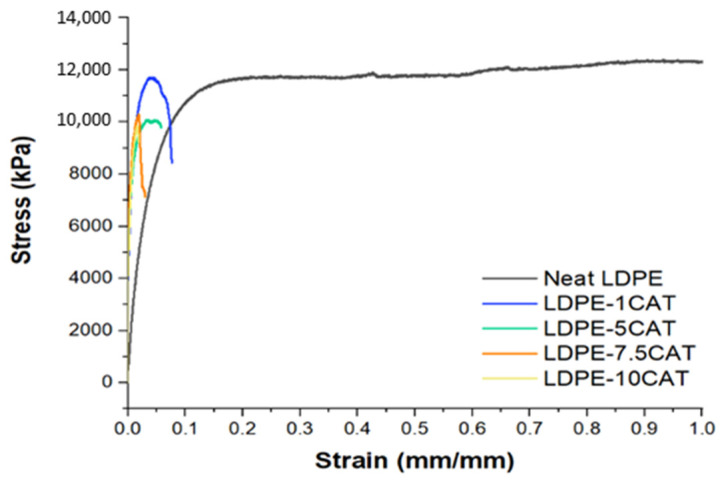
Representative stress–strain curves of neat LDPE and LDPE–CAT blends.

In Figure 8, representative tensile test curves for neat LDPE and LDPECAT samples are presented, while Table 3 reports the main calculated parameters, including tensile modulus, yield stress, yield strain, and elongation at break. Neat LDPE shows a high elongation capacity, exceeding 100%. However, the presence of CAT in the blend causes a sharp reduction in maximum elongation at break, decreasing to 27% at 1 wt% CAT and only 8% at 10 wt% CAT. The stiffness of the blend increases proportionally to the amount of CAT present, with a maximum increase in the tensile modulus of 37% at 10 wt% CAT with respect to neat LDPE [44,45]. Nevertheless, yield strength and strain are not improved, showing no change at 1 wt% CAT, and a 17% reduction at higher CAT content.

These results are likely due to the incompatibility between LDPE and CAT polymers, which is responsible for the generation of small CAT domains during extrusion, as demonstrated by SEM analysis. The higher stiffness of the CAT polymer compared to LDPE increases the overall elastic modulus, but once the matrix undergoes plastic deformation, the reduced interface strength between the two phases causes early breaking and crack development, weakening the blend through a continuous formation of breaks. As a result, the blend’s capability to withstand large deformations is decreased, and both yield stress and strain are lowered.

## 4. Conclusions

In this study, the microstructural, chemical, thermal, rheological, and mechanical properties of blends based on LDPE with different contents of a bioplastic made from cellulose acetate plasticized with triacetin (CAT) were evaluated. The effect of the presence of CAT resulted in evident features seen in SEM images, where the immiscibility of the blends was clearly observed by the presence of two separated phases. The FTIR analyses confirmed the incompatibility between the two components, with the formation of a new peak at 1556 cm^−1^ in the ATR spectra of blends indicating the presence of the carboxylate group. This peak could not be attributed to the formation of specific interactions between CAT and LDPE but can be considered a consequence of the thermal treatment (extrusion) of the LDPE in the blends, as reported in the literature. In addition, FTIR analysis highlighted that there was no degradation of neat LDPE, and the addition of CAT in various proportions did not encourage the degradation of LDPE after one cycle of extrusion. The thermal stability of LDPE slightly increased in the blends, while that of CAT decreased. The addition of CAT resulted in a reduction in the degree of crystallinity of the LDPE–CAT blends. Moreover, the addition of CAT decreased viscosity, yield stress, yield strain, and elongation at break, while increased the tensile modulus, with the most significant parameters changes in the blend with 10 wt% CAT. Then, the results of this research suggest that the presence of CAT does not significantly affect the chemical, thermal, and rheological properties of LDPE, but has a negative impact on the processability and mechanical behavior of LDPE, resulting in reduced quality of the recycled material, which cannot be used for certain types of application.

As a general conclusion, it is best to avoid adding cellulose-based bioplastics to the plastic waste stream since they will strongly affect the recycling of sorted LDPE packaging waste. The presence of bioplastic in the LDPE packaging stream can also have some economic implications. For instance, in Italy, the consortium for the plastic packaging waste management (namely COREPLA) limits the presence of bioplastics items in LDPE streams sorted in MRF for a value between 5.5 and 7.0%. If bioplastics exceed these limits, the MRF has to pay an expensive fee. As the presence of bioplastics in such streams does not exceed 3% [15], the current amount of bioplastics will not generate a significant economic burden. In contrast, keeping in mind the growing trend of bioplastics presence in the entire municipal solid waste stream, this could happen in the near future. Therefore, MRFs should create a separate sorting stream for bioplastic waste in order to reduce the contamination of these materials on the sorted products.

## Figures and Tables

**Table 1:** Thermal stability of neat LDPE and LDPE-10CAT.

Sample	T_dmax,1_ [°C]	T_dmax,2_ [°C]
CAT	369	-
Neat LDPE	-	440
LDPE-10CAT	336	443

T_dmax_, maximum decomposition temperature.

**Table 2:** Melting temperature (Tm), crystallization temperatures (Tc_1_ and Tc_2_), crystallization enthalpies (ΔHc_1_ at Tc_1_ and ΔHc_1_ at Tc_2_) and degree of crystallinity (Xc) for neat LDPE and LDPE–CAT blends.

Sample	T_m_ [°C]	T_c1_ [°C]	T_c2_ [°C]	ΔHc_1_ [J/g]	ΔHc_2_ [J/g]	X_c_ [%]
Neat LDPE	116	90	58	77	5.1	27.8
LDPE-1CAT	110	96	60	74	4.2	27.4
LDPE-5CAT	110	96	60	75	4.3	27.1
LDPE-7.5CAT	110	96	60	74	4.1	26.7
LDPE-10CAT	110	96	60	75	4.2	26.7

**Table 3:** Calculated values of tensile parameters for neat LDPE and LDPE–CAT blends (mean and deviation).

Sample	Tensile Modulus [MPa]	Yield Stress [MPa]	Yield Strain [mm/mm]	Elongation at Break [mm/mm]
Neat LDPE	248.7 ± 6.9	11.9 ± 0.1	0.24 ± 0.00	1.1 ± 0.04
LDPE-1CAT	265.8 ± 15.6	12.0 ± 0.5	0.16 ± 0.01	0.43 ± 0.06
LDPE-5CAT	280.3 ± 11.1	10.3 ± 0.2	0.17 ± 0.01	0.37 ± 0.05
LDPE-7.5CAT	308.3 ± 23.5	10.0 ± 0.6	0.12 ± 0.02	0.28 ± 0.05
LDPE-10CAT	341.5 ± 49.0	9.0 ± 0.9	0.11 ± 0.03	0.16 ± 0.05

## Data Availability

Not applicable.
